# CMR shows that anthracycline cardiotoxicity is common in women treated for early breast cancer and associated with undiagnosed hypertension; but cannot be reliably detected using late-gadolinium enhancement imaging

**DOI:** 10.1186/1532-429X-15-S1-P276

**Published:** 2013-01-30

**Authors:** Paul Kotwinski, Gillian Smith, Julie Sanders, Jackie Cooper, David Kotwinski, Albert Teis, Michael (Monty) G Mythen, Alison Jones, Hugh E Montgomery, Dudley J Pennell

**Affiliations:** 1 Institute of Human Health and Performance, UCL, London, UK; 2 CMR Unit, The Royal Brompton Hosptial, London, UK; 3 Department of Oncology, UCL Partners, London, UK; 4 Centre for Cardiovascular Genetics, UCL, London, UK; 5 Biomedical Research Unit, UCLH/UCL, London, UK

## Background

A growing number of patients with cancer are at risk from chronic anthracycline cardiotoxicity (cAC) as a result of improving cancer prognosis. Although susceptibility is cumulative dose-related, it is also idiosyncratic. Furthermore, at present there is no way to reliably identify those at risk. The common practice of serially measuring LV ejection fraction (LVEF) only identifies cardiotoxicity after significant damage has been incurred. We hypothesised that risk of cAC could be determined from patient and treatment factors, known at baseline, together with assessment of the cardiac response to the 1st dose of anthracycline. Here we report the prevalence of cAC detected using CMR, together with the associations with baseline BP and anthracycline dose.

## Methods

Women due to receive anthracycline-based chemotherapy for early breast cancer were recruited. Those with known cardiovascular disease were excluded. CMR was performed on Siemens 1.5T scanners before chemotherapy and at follow-up (>1 year after the final anthracycline cycle, and >3 months after Trastuzumab). LVEF was measured from cine images by a single operator (PK) blind to clinical and temporal data, using CMRtools. Chronic AC (cAC) was defined as a fall in absolute LVEF ≥5%.

## Results

164 subjects completed the study 18.5 [15.1-23.7] months after finishing treatment. 99% received epirubicin (400 [300-450] mg/m2). The mean fall in LVEF was 2.2% (p<0.001), compared with a projected increase of 0.2%. The cardiac response was heterogeneous: 20.7% (N=34) were in the subclinical cAC group. Mean LVEF also fell in the remainder of the cohort (by 0.8%, p=0.03), demonstrating this group were minimally affected, rather than unaffected. No subjects developed late gadolinium enhancement as a result of treatment (LGE images in N=119). There was a trend towards patients who received epirubicin ≥450mg/m2 being in the cAC group (odds ratio (OR) 2.12 [0.97-4.60], p=0.06). Patients with baseline BP ≥140/90mmg were more likely to be in the cAC group (OR 3.63 [1.31-10.09], p=0.01); of these 18, only 1 was diagnosed and treated for hypertension.

## Conclusions

Falls in LVEF ≥5% were detected in >20% of patients. Declines in resting LVEF are relatively late in the pathological process of cAC and therefore likely to reflect increased risk of heart failure as a ‘late-effect' of cancer treatment. Our data suggest that undiagnosed hypertension was common; diagnosing and treating it may offer an opportunity to reduce cAC. A fall in LVEF ≥5% detected by CMR may be a better end-point for monitoring cardiotoxicity in oncology trials than those currently used. Although fibrosis is a hallmark of cAC based on biopsy and ex-vivo data, it cannot be reliably detected using LGE; T1-mapping may be of greater utility.

## Funding

This abstract presents independent research funded by the UK Department of Health under the Genetics Health Research programme. It was sponsored by University College London (UCL), and supported by the cardiovascular Biomedical Research Unit of Royal Brompton Hospital and Imperial College. Patients were recruited through the auspices of the National cancer research Network (NCRN). The views expressed in this publication are those of the author(s) and not necessarily those of the NHS, NCRN or the Department of Health.

